# Antigen Presenting Cells Contribute to Persistent Immune Activation Despite Antiretroviral Therapy Initiation During Hyperacute HIV-1 Infection

**DOI:** 10.3389/fimmu.2021.738743

**Published:** 2021-09-24

**Authors:** Kewreshini K. Naidoo, Okechukwu C. Ndumnego, Nasreen Ismail, Krista L. Dong, Thumbi Ndung’u

**Affiliations:** ^1^ HIV Pathogenesis Programme, Doris Duke Medical Research Institute, Nelson R. Mandela School of Medicine, University of KwaZulu-Natal, Durban, South Africa; ^2^ Africa Health Research Institute, Durban, South Africa; ^3^ Females Rising Through Education, Support and Health, Durban, South Africa; ^4^ Ragon Institute of Massachusetts General Hospital, Massachusetts Institute of Technology, and Harvard University, Cambridge, MA, United States; ^5^ Max Planck Institute for Infection Biology, Berlin, Germany; ^6^ Division of Infection and Immunity, University College London, London, United Kingdom

**Keywords:** antigen presenting cells, immune activation, inflammation, hyperacute HIV-1 infection, antiretroviral therapy

## Abstract

Human immunodeficiency virus (HIV)-induced changes in immune cells during the acute phase of infection can cause irreversible immunological damage and predict the rate of disease progression. Antiretroviral therapy (ART) remains the most effective strategy for successful immune restoration in immunocompromised people living with HIV and the earlier ART is initiated after infection, the better the long-term clinical outcomes. Here we explored the effect of ART on peripheral antigen presenting cell (APC) phenotype and function in women with HIV-1 subtype C infection who initiated ART in the hyperacute phase (before peak viremia) or during chronic infection. Peripheral blood mononuclear cells obtained longitudinally from study participants were used for immunophenotyping and functional analysis of monocytes and dendritic cells (DCs) using multiparametric flow cytometry and matched plasma was used for measurement of inflammatory markers IL-6 and soluble CD14 (sCD14) by enzyme-linked immunosorbent assay. HIV infection was associated with expansion of monocyte and plasmacytoid DC (pDC) frequencies and perturbation of monocyte subsets compared to uninfected persons despite antiretroviral treatment during hyperacute infection. Expression of activation marker CD69 on monocytes and pDCs in early treated HIV was similar to uninfected individuals. However, despite early ART, HIV infection was associated with elevation of plasma IL-6 and sCD14 levels which correlated with monocyte activation. Furthermore, HIV infection with or without early ART was associated with downmodulation of the co-stimulatory molecule CD86. Notably, early ART was associated with preserved toll-like receptor (TLR)-induced IFN-α responses of pDCs. Overall, this data provides evidence of the beneficial impact of ART initiated in hyperacute infection in preservation of APC functional cytokine production activity; but also highlights persistent inflammation facilitated by monocyte activation even after prolonged viral suppression and suggests the need for therapeutic interventions that target residual immune activation.

## Introduction

In the absence of antiretroviral therapy, infection with human immunodeficiency virus type 1 (HIV-1) is characterised by prolonged viremia, progressive CD4+ T cell loss, and persistent immune activation that ultimately leads to compromised immune responses. Combination antiretroviral therapy (cART) is successful in supressing viral replication and is usually administered following HIV-1 diagnosis during the chronic phase of infection. However, despite the benefits of improving the quality of life of people living with HIV, current therapy requires life-long, uninterrupted adherence. Furthermore, chronic immune activation and inflammation persist in these individuals which translates into higher risk of coinfections and comorbidities (1, 2), and moreover, long-term viral suppression does not necessarily equate to full immune restoration (3). Reconstitution of host immunity and reduction of HIV-associated comorbidities are highly dependent on the timing of cART commencement (4, 5). Initiation of therapy as close as possible to the time of infection is not only clinically beneficial but it may also lead to post treatment control of viremia following antiretroviral therapy interruption in a subset of individuals (6, 7). Therefore, a better understanding of the impact of treatment of acute HIV infection before or around peak viremia is needed to develop new interventions for preservation or restoration of immunity and to inform HIV cure or post-treatment control strategies in the absence of antiretroviral therapy.

Commencement of cART in Fiebig stage I-II blunts peak viremia, preserves CD4 T cell counts, prevents seroconversion, limits the size and anatomic spread of viral reservoirs and averts loss of mucosal Th17 cell counts and cytokine polyfunctionality (8–14). Treatment of hyperacute HIV infection also results in pro-inflammatory cytokines and soluble plasma biomarker profiles that resemble pre-infection levels, although some persistent immune activation has also been reported (15–18). Moreover, early treatment prevents the development of defective adaptive immunity, with reduced T and B cell activation and enhanced functionality compared to treatment-naïve infection (17, 19–21). However, the impact of early cART on innate immune mechanisms is less established.

Antigen presenting cells (APCs) – monocytes and dendritic cells (DCs) – are integral to the development of robust innate and adaptive immunity immediately following infection. In addition to their role in antigen presentation, APCs are the main source of type I interferons (IFNs) which mobilise intracellular antimicrobial pathways and orchestrate innate and adaptive immune development (22). Type I IFNs and other pro-inflammatory cytokines play a complex role in mediating antiviral immunity, where a temporal burst of IFN-I secretion is thought to be advantageous over prolonged production with the latter contributing to clinically detrimental immune activation and systemic inflammation (23–27). Although cART suppresses circulatory type I IFNs, some levels still persist and upregulation of interferon-stimulated genes (ISGs) in peripheral blood cells and lymphoid organs have been reported (28, 29). Our group recently described the dynamics of pro-inflammatory markers in the earliest stages of infection. Several cytokines (including IFN-α) that usually contribute to the cytokine storm in untreated acute HIV infection (AHI) were similar or only moderately elevated compared to pre-infection levels when ART was started prior to or at peak viremia. However, even with early viral suppression, soluble CD14 (sCD14) remained elevated above physiological levels during AHI and did not resolve by chronic phase infection. Moreover, Fiebig stage I-II HIV infection was associated with dysregulation of particular lymphoid and myeloid cell populations even prior to commencement of cART (16). Collectively, these data suggest differential effects of early ART on the activation kinetics or dysregulation of immune cells, which could potentially have long-term clinical consequences.

Here, our goal was to gain further insights into immunological processes that may be altered by HIV infection, with or without antiretroviral treatment initiated during Fiebig stages I-II (before peak viremia). Specifically, we investigated frequency, activation and functional dynamics of the major APCs (monocytes, myeloid DCs and plasmacytoid DCs), building on our earlier studies that suggested innate immune cell expansion and activation during the hyperacute phase of infection even before cART initiation. Participants were longitudinally followed, comparing those who initiated treatment during the hyperacute phase of infection with those who initiated treatment approximately a year post-infection. In this pilot study we provide evidence of prolonged activation of innate immunity, particularly monocytes, in the absence of T cell activation, which may contribute to ongoing inflammation and other immune dysfunction despite therapy intervention before peak viremia. Furthermore, we show that there is impaired pDC functionality in untreated individuals from as early as 1-month post-infection, whereas responses to antigenic stimulation are preserved at pre-infection levels with early ART commencement.

## Materials and Methods

### Study Participants and Staging of HIV-1 Infection

Participants were 18-23 year old females from the Females Rising through Education Support and Health (FRESH) cohort, an ongoing longitudinal study based in Durban, KwaZulu-Natal, South Africa (10, 30). Participants at high risk of HIV-1 infection consent to twice-weekly finger-prick blood sampling for HIV-1 RNA testing to enable detection of HIV infection during acute HIV infection (AHI). Biological samples are collected pre and post-infection. At study inception, the study protocol adhered to the South African national HIV clinical management guidelines, which restricted ART initiation to CD4 counts at or below 350 cells/mm^3^. Accordingly, participants testing HIV-1 RNA positive during this period did not initiate therapy until they met eligibility criteria. Approval to initiate ART during AHI was obtained, allowing for immediate treatment upon detection of HIV-1 RNA. Participation was voluntary and all participants provided written informed consent in accordance with protocols approved by the Biomedical Research Ethics Committee of the University of KwaZulu-Natal and the Institutional Review Board for Massachusetts General Hospital. In this sub-study, twenty-seven individuals from the FRESH cohort were included according to HIV status and time of ART initiation as follows: HIV-1 negative (n=10), HIV positive who initiated ART at the hyperacute phase (n=10), and HIV positive who initiated ART during chronic infection (n=7). HIV positive individuals, either starting ART at hyperacute (Fiebig stage I-II) or chronic infection were assessed at 1- and 12- months post-infection. Fiebig stage I-II was characterised as HIV-1 RNA positive (NucliSENS EasyQ HIV-1 v2.0 kit, BioMérieux, Marcy-l’Étoile, France), p24 antigen negative or positive and HIV antibody rapid or ELISA assay negative (Elecsys HIV combi PT 4th Generation (Ag+Ab test) p24 Ag, Roche, Basel, Switzerland) and Western blot negative (GS HIV-1 Western blot kit, Bio-Rad Laboratories, Hercules, CA, USA). Individuals starting ART during chronic infection were treatment naïve at the 1- and 12-month post-infection time points. These individuals subsequently initiated ART and were further assessed at 12 months after starting ART (i.e., approximately 24-months post-infection). Samples for the participants initiating ART during chronic infection were limited at the 1- month (n=4) and 24-month (n=6) time points.

### Clinical Parameters

Blood samples were collected in ethylenediaminetetraacetic acid (EDTA) anticoagulated vacutainer tubes (Becton Dickinson (BD), Franklin Lakes, NJ, USA) for CD4+ T cell count measurement and viral load quantification. CD4 counts were measured using BD Trucount and analysed on a four-parameter FACSCalibur flow cytometer (BD). Viral loads were determined using the NucliSENS EasyQ HIV-1 v2.0 kit with a detection limit of 20 copies/ml (BioMérieux, Marcy-l’Étoile, France). Individuals initiating ART were prescribed a fixed-dose three drug oral combination pill of efavirenz, emtricitabine and tenofovir. For participants who started antiretroviral therapy in acute HIV infection, raltegravir was prescribed as a fourth drug until 90 days after viral suppression.

### Antibodies and Reagents

Monoclonal antibodies were obtained from BD Biosciences: fluorescein isothiocyanate (FITC) anti-human CD8 (RPA-T8), phycoerythrin (PE)-CF594 anti-human HLA-DR (G46-6), PE-Cyanine(Cy)7 anti-human CD86 (FUN-1), PE anti-human IFN-α[2b] (7N4-1) and BioLegend (San Diego, CA, USA): brilliant violet (BV) 650 anti-human CD3 (OKT3), BV510 anti-human CD19 (HIB19), BV510 anti-human CD56 (NCAM) (HCD56), allophycocyanin (APC) anti-human CD4 (SK3), BV785 anti-human CD16 (3G8), APC-Cy7 anti-human CD14 (HCD14), PE-Cy5 anti-human CD11c (3.9), BV421 anti-human CD123 (6H6), BV711 anti-human CD38 (HIT2), peridinin-chlorophyll-protein (PerCP)/Cy5.5 anti-human CD69 (FN50), BV605 anti-human TNF-α (MAb11). Anti-Mouse Ig, κ/Negative control particles were used for flow cytometry compensation (BD Biosciences). LIVE/DEAD Fixable Aqua dead cell stain kit and FIX & PERM cell permeabilization kit were sourced from Invitrogen (Carlsbad, CA, USA). Toll-like receptor (TLR) agonists for cell stimulation were as follows: TLR4 ligand lipopolysaccharide (LPS) was from Sigma-Aldrich (Saint Louis, MO, USA), TLR7/8 ligand imidazoquinoline compound (CL097) and TLR9 ligand class A CpG oligonucleotide (ODN 2216) were from InvivoGen (San Diego, CA, USA). The DOTAP liposomal transfection reagent was sourced from Roche. Brefeldin A (BFA) was obtained from Sigma-Aldrich. R10 medium was Roswell Park Memorial Institute (RPMI) 1640 supplemented with 10% gamma irradiated, heat-inactivated foetal bovine serum (Gibco, Waltham, MA, USA), 1% penicillin/streptomycin, 1% L-glutamine and 1% HEPES buffer (Lonza, Basel, Switzerland).

### Immunophenotyping and Intracellular Cytokine Staining

Blood samples were collected in acid citrate dextrose (ACD) vacutainer tubes (BD). Peripheral blood mononuclear cells (PBMCs) were isolated by Ficoll density gradient centrifugation and cryopreserved within 6 hours until use. Cryopreserved PBMCs were thawed, re-suspended in R10 medium and rested at 37°C and 5% CO_2_ for at least 2 hours. For immunophenotyping, one million cells were stained with LIVE/DEAD Fixable Aqua dead cell stain and monoclonal antibodies for CD19 (to exclude B cells), CD56 (to exclude NK cells), CD3, CD4, CD8 (to identify T cells), CD11c, CD14, CD16, CD123 and HLA-DR (to identify monocytes, myeloid dendritic cells [mDCs] and plasmacytoid dendritic cells [pDCs]) and activation and co-stimulatory receptors CD38, CD69 and CD86, before fixation with Medium A and acquisition on a BD LSRFortessa. For *in vitro* functional assays: monocytes, mDCs and pDCs were assessed for cytokine production (TNF-α and IFN-α) by culturing one million PBMCs in either R10 medium alone, TLR4 ligand LPS, TLR7/8 ligand CL097, or TLR9 ligand CpG in the presence of DOTAP. BFA was added to all conditions at a final concentration of 10 μg/ml. Following overnight culture at 37°C and 5% CO_2_, samples were stained with LIVE/DEAD Fixable Aqua dead cell stain, CD3, CD11c, CD14, CD16, CD19, CD56, CD123 and HLA-DR antibodies (as described above), fixed with Medium A, before permeabilization with Medium B and staining with monoclonal antibodies for TNF-α and IFN-α, and acquisition on a BD LSRFortessa.

### Enzyme-Linked Immunosorbent Assay

Blood samples were collected in EDTA anticoagulated vacutainer tubes. Plasma component was separated by centrifugation within 6 hours followed by immediate storage at -80°C until use. Plasma concentrations for IL-6 were measured using Human IL-6 Quantikine ELISA Kit (R&D Systems, Minneapolis, Min, USA). sCD14 was measured using Human CD14 DuoSet ELISA (R&D Systems).

### Software and Statistical Analysis

FACSDiva 7 (BD) was used for flow cytometry data acquisition. FlowJo software version 9 (FlowJo, LLC, Ashland, OR, USA) was used for flow cytometry data analysis. GraphPad Prism 5 (GraphPad software Inc., La Jolla, CA, USA) was used for statistical data analysis. Mann-Whitney *U* test was used for single group comparisons. Kruskal-Wallis test was used for multiple comparisons followed by Bonferroni’s correction test for multiple comparisons. Correlations were examined using Spearman rank analysis and p-values <0.05 were considered statistically significant. All statistically significant differences are marked with the p-value in the figures and non-significant differences are unmarked.

## Results

Participants in this study were HIV negative (n=10), HIV positive initiated on ART during Fiebig I-II (ART hyperacute) (n=10) and HIV positive initiated on ART during chronic infection (ART chronic) (n=7). For the chronic treated group only 4 samples at 1-month and 6 samples at 24-months were available. Participant demographic and clinical characteristics are summarised in **Table 1**.

**Table 1:** Clinical parameters of study participants.

Characteristic	Participant groups						ANOVA p value
	HIV- (n = 10)	HIV+ (n = 10)		HIV+ (n = 7)			
		ART initiation: hyperacute		ART initiation: chronic			
Time post-infection	na	1 month	12 months	1 month*	12 months	24 months*	na
Time post ART initiation	na	1 month	12 months	naïve	naïve	12 months	na
Age at study initiation, years	21	18			22		0.773
	(20-22)	(18-22)			(20-24)
CD4 count, cells/mm^3^	1045	848	907	564	490	574	<0.0001
	(823-1433)	(723-988)	(815-977)	(522-636)	(305-601)	(504-694)
Viral load, RNA copies/ml	na	<20	<20	107,250	82,000	<20	<0.0001
				(3,975-262,500)	(3,500-150,000)	

Data is represented as median (IQR). *PBMC samples were available for only 4 participants at 1 month and for 6 participants at 24 months post-infection in the group that initiated ART during chronic infection. All participants are female. Comparisons of age, CD4 count and viral load between participant groups were determined by one-way ANOVA (Kruskal-Wallis test).

### Viral Suppression, CD4 Count Maintenance, and Controlled T Cell Activation Following ART Initiation During Hyperacute HIV-1 Infection

**Figure 1:**
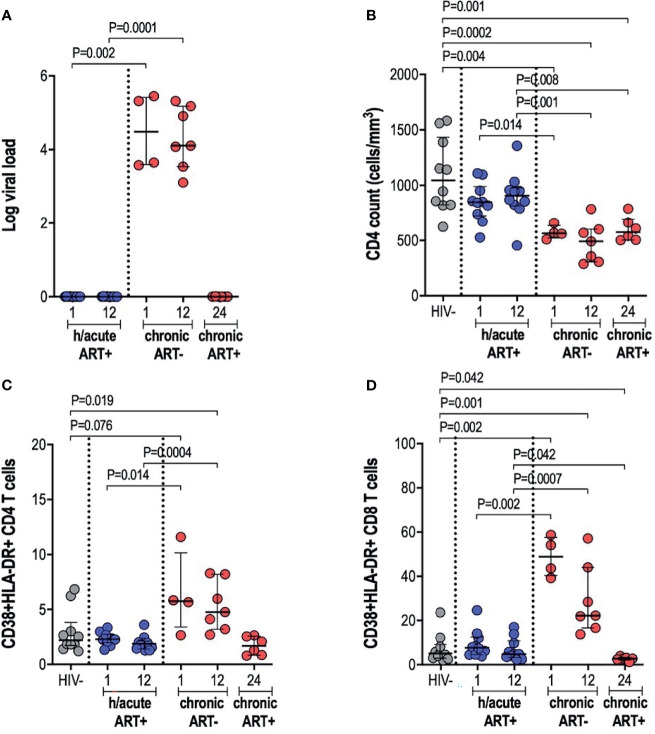
Log viral load, CD4 T cell counts and T cell activation. **(A)** Log viral load in HIV infected participants. **(B)** CD4+ T cell count. **(C)** CD4+ T cell activation and **(D)** CD8+ T cell activation in HIV negative individuals and hyperacute and chronic ART groups at month 1, 12 and 24 post-infection.

Early treated individuals achieved undetectable viremia (<20 RNA copies/ml) within 1-month post-infection, and suppression was maintained throughout the first year of infection, whilst those treated during chronic infection had a median viral load of 107,250 and 82,000 RNA copies/ml at 1- and 12-months respectively (**Table 1** and **Figure 1A**). CD4+ T cell counts differed significantly across the 3 study groups and by time points analysed (**Table 1** and **Figure 1B**). Median CD4+ T counts for individuals treated with ART during hyperacute infection were 848 and 907 cells/mm^3^ at 1- and 12-months respectively, which was not significantly different from the median count of 1,045 cells/mm^3^ for HIV uninfected individuals (**Figure 1B**). In contrast, individuals initiated on ART during chronic infection presented with CD4+ T cell decline, with counts significantly lower compared to the uninfected controls at both 1-month (p=0.004) and 12-months (p=0.0002) post-infection. Individuals initiating treatment during chronic infection also had significantly lower CD4+ T cell counts compared to hyperacute treated individuals at 1-month (p=0.014) and at 12-months (p=0.001) post-infection. After 12 months on ART (24-months post-infection), CD4 counts in ART chronically treated individuals remained significantly lower compared to HIV uninfected participants (p=0.001) and the ART hyperacute treated group at 12-months (p=0.008) (**Figure 1B**). We further evaluated the activation profiles of CD4+ and CD8+ T cells by assessing CD38 and HLA-DR co-expression (**Figures 1C, D** and **Supplementary Figure 1**). ART hyperacute treated individuals displayed similar frequencies of activated CD4+ and CD8+ T cells compared to HIV negative individuals (**Figures 1C, D**). Higher CD4+ T cell activation was observed in the ART chronic phase treated group (before therapy initiation) at 1-month (p=0.076) and at 12-months (p=0.019) compared to HIV negative individuals. Chronic phase treated persons also displayed higher CD4+ T cell activation at 1-month (p=0.014) and 12-months (p=0.0004) compared to those initiating treatment during the hyperacute phase (**Figure 1C**). Following initiation of ART in the chronically treated group, CD4+ T activation reduced such that the frequency of activated cells was not statistically different to that in the HIV uninfected and ART hyperacute treated groups (**Figure 1C**). Similarly, chronically treated individuals displayed higher CD8+ T activation at 1-month (p=0.002) and at 12-months (p=0.001) compared to HIV negative participants (**Figure 1D**). Moreover, CD8+ T cell activation was higher in chronic phase treated individuals compared to ART hyperacute phase treated participants at 1-month (p=0.002) and at 12-months (p=0.0007) post-infection (**Figure 1D**). Following 12 months of therapy for the chronically treated individuals, activated CD8+ T cell frequencies declined and were significantly lower than in HIV negative and ART hyperacute treated individuals (p=0.042 for both comparisons) (**Figure 1D**). Overall, these results indicate that ART during the hyperacute phase of HIV infection prevented immune activation of the CD4+ and CD8+ T cell populations, and that initiation of ART during the chronic infection phase reduced immune activation to baseline pre-infection levels.

### Elevated Monocyte and pDC Frequencies in Early Treated Individuals With Distinct Monocyte Subset Distribution

**Figure 2:**
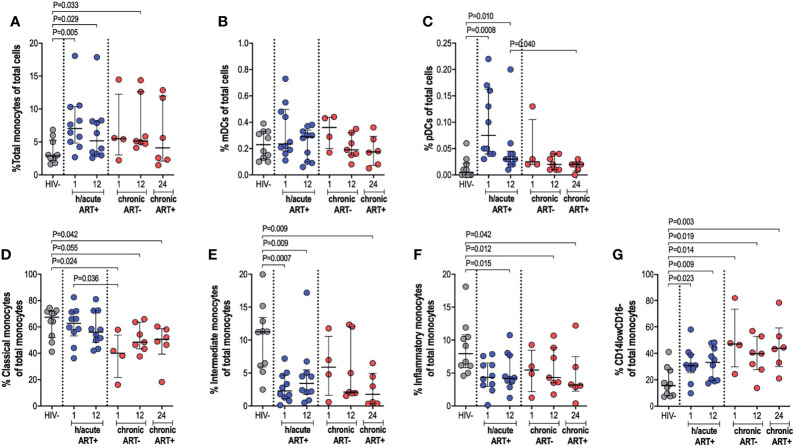
Antigen presenting cell frequencies. HIV negative individuals and hyperacute and chronic ART groups at month 1, 12 and 24 post-infection frequencies of **(A)** monocytes, **(B)** mDCs and **(C)** pDCs as a percentage of total leukocytes. Monocytes were further classified as **(D)** classical, **(E)** intermediate, **(F)** inflammatory or **(G)** CD14lowCD16- as a percentage of total monocytes by relative expression of CD14 and CD16.

The impact of very early ART initiation on phenotypic and functional characteristics of professional APCs is not well characterised and yet this may have implications for the maintenance of optimal immunity following infection and for HIV cure or remission strategies. To address this, we performed *ex vivo* flow cytometry analysis of monocytes, mDCs and pDCs in individuals initiating therapy during Fiebig stage I-II in contrast to individuals initiating therapy in the chronic phase of infection and compared these groups to HIV negative individuals. We first assessed monocyte, mDC and pDC cell frequencies; and then further categorised monocytes as classical, intermediate or inflammatory based on CD14 and CD16 expression (**Supplementary Figure 2**). Cell types were enumerated as a percentage of total cells. Monocyte frequencies were noticeably elevated in the ART hyperacute treated compared to the uninfected group, particularly in acute phase infection (p=0.005 at 1-month, p=0.029 at 12-months, **Figure 2A**). Likewise, median monocyte frequencies were seemingly elevated in the ART chronically treated group at 1-month post-infection and were significantly higher at 12-months post-infection (p=0.033) compared to uninfected individuals (**Figure 2A**). mDC frequencies were not affected by HIV or ART intervention with no significant differences noted in median cell frequencies between the groups irrespective of HIV infection status or ART initiation timing (**Figure 2B**). pDCs were drastically elevated at 1-month post-infection (p=0.0008), with partial decline in frequencies by 12-months post-infection (p=0.01) for the hyperacute phase treated group (**Figure 2C**). In patients initiating treatment during chronic infection, pDC frequencies appeared elevated at 1-month post-infection and declined thereafter with no significant difference noted at 12-months post-infection when the individuals were treatment naïve and at 24-months post-infection (i.e. 12-months post-ART initiation). In fact, pDC frequencies were significantly higher at 12-months post-ART in the hyperacute treated group compared to 24-months for the chronic treated group (i.e. 12-months post-ART initiation) (p=0.04, **Figure 2C**). We further investigated monocyte subsets – classical, intermediate and inflammatory monocytes were classified according to common nomenclature (31). Classical subset frequencies following early ART were maintained at levels that resembled uninfected individuals. In contrast, lower frequencies compared to uninfected controls were observed in the group initiating treatment in the chronic phase (p=0.024 at 1-month, p=0.055 at 12-months) and even after ART commencement at 24-months post-infection (p=0.042) (**Figure 2D**). Intermediate monocytes were significantly lower in treated groups regardless of ART timing (p=0.0007 at 1-month, p=0.009 at 12-months in ART hyperacute; p=0.009 at 24-months in ART chronic, **Figure 2E**), whilst reduced inflammatory frequencies were linked to infection duration rather than ART status or timing (p=0.015 at 12-months in ART hyperacute; p=0.012 at 12-months, p=0.042 at 24-months in ART chronic, **Figure 2F**). Interestingly, we observed a distinct CD14lowCD16- phenotype, which was considerably higher amongst HIV infected groups, irrespective of ART initiation or length of infection (**Figure 2G** and **Supplementary Figure 2**). Overall, these data suggest that early initiation of antiretroviral therapy does not always prevent immunological changes in APC populations, with remarkable heterogeneity linked to HIV and ART initiation on different APC cell populations.

### Sustained Expression of Activation Marker CD69 but Downregulated Expression of Co-Stimulatory Marker CD86 on APCs Following Early ART

**Figure 3:**
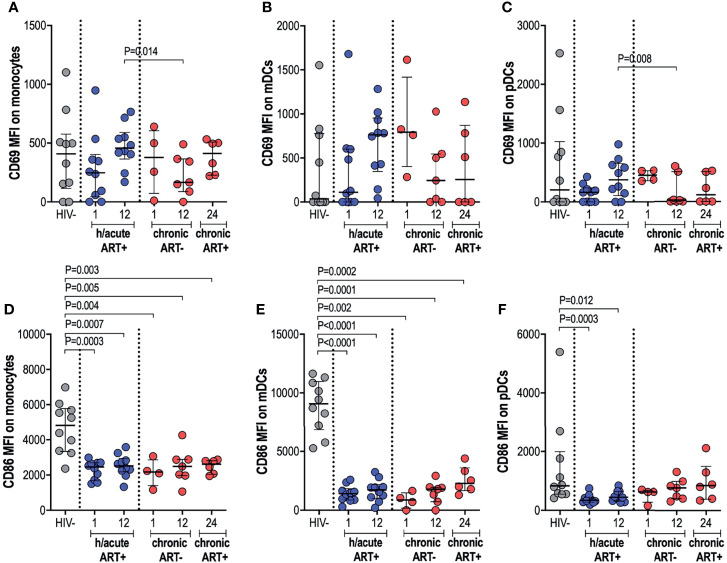
CD69 and CD86 expression on antigen presenting cells. HIV negative individuals and hyperacute and chronic ART groups at month 1, 12 and 24 post-infection were assessed for expression of activation marker CD69 on **(A)** monocytes, **(B)** mDCs and **(C)** pDCs and co-stimulatory marker CD86 on **(D)** monocytes, **(E)** mDCs and **(F)** pDCs.

Next, we measured the *ex vivo* expression of inducible receptors CD69 and CD86 on APCs (**Supplementary Figure 3**). Early treated individuals exhibited a non-significant dip in CD69 expression on monocytes, compared to HIV uninfected persons, with a recovery to pre-infection levels by 12-months post-infection (**Figure 3A**). In participants who initiated treatment in the chronic phase, CD69 expression on monocytes was decreased at 12-months post-infection compared to early treated individuals (p=0.014) but with recovery at 12-months post-ART initiation (**Figure 3A**). CD69 expression on mDCs was highly variable from participant to participant with no significant differences between the groups noted (**Figure 3B**). CD69 expression on pDCs in hyperacute treated individuals was similar to uninfected controls but levels were significantly lower at 12-months post-infection in participants starting ART in chronic HIV-1 infection when compared to hyperacute treated persons (p=0.008), with partial recovery after ART initiation (**Figure 3C**). Assessment of the co-stimulatory maker CD86 on APCs revealed significantly lower expression levels on monocytes and mDCs in all HIV-infected groups compared to HIV uninfected individuals (**Figures 3D, E**). Interestingly, CD86 expression on pDCs was only downregulated in the ART hyperacute group (p=0.0003 at 1-month, p=0.012 at 12-months, **Figure 3F**), while ART chronic groups displayed no differences in pDC CD86 expression compared to uninfected individuals. Taken together, these results indicate differential impact of hyperacute ART on APCs such that whereas it may prevent the downregulation of CD69 on monocytes and pDCs, it does not prevent downmodulation of CD86 on monocytes and mDCs, and it may lead to downmodulation of CD86 on pDCs that does not appear to be HIV-1 infection driven.

### Elevated IL-6 and sCD14 Levels Despite Early ART

**Figure 4:**
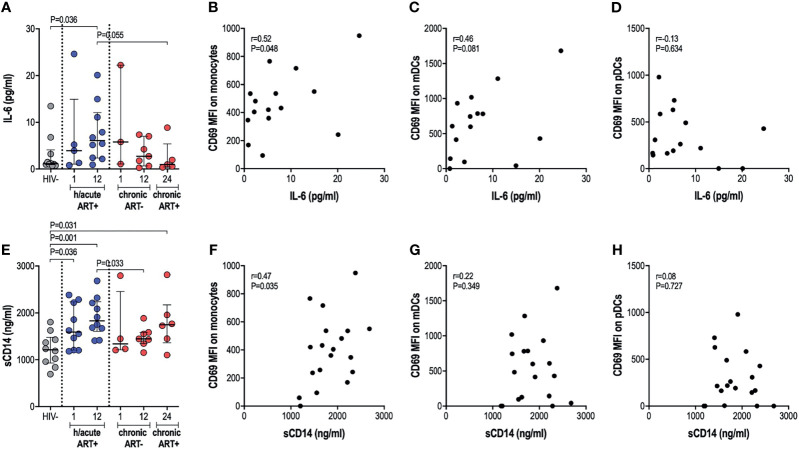
Plasma IL-6 and soluble CD14 levels. **(A)** Plasma IL-6 in HIV negative individuals and hyperacute and chronic ART groups at month 1, 12 and 24 post-infection. Correlations between plasma IL-6 and CD69 expression on **(B)** monocytes, **(C)** mDCs **(D)** pDCs in the hyperacute ART group at month 1 and 12 post-infection. **(E)** Soluble CD14 in HIV negative individuals and hyperacute and chronic ART groups at month 1, 12 and 24 post-infection. Correlations between soluble CD14 and CD69 expression on **(F)** monocytes, **(G)** mDCs and **(H)** pDCs in the hyperacute ART group at month 1 and 12 post-infection. Associations show the rho value of Spearman correlation.

We next assessed whether cellular activation of APCs, sometimes noted despite ART initiation during hyperacute infection, translated into systemic inflammation considering that the latter is commonly implicated in disease progression to AIDS. We therefore investigated the effect of early therapy on IL-6 and sCD14 circulatory levels. No significant differences in IL-6 levels were found in untreated infection compared to uninfected individuals. Surprisingly, early treated individuals exhibited significantly higher IL-6 levels at 12-months post-infection (p=0.036, **Figure 4A**). Late treated individuals trended towards lower levels of IL-6 after one year on ART compared to the early treatment group (p=0.055, **Figure 4A**). IL-6 positively associated with expression of activation marker CD69 on monocytes (rho=0.52, p=0.048; **Figure 4B**) and mDCs (rho=0.46, p=0.081; **Figure 4C**); whereas no correlation was found between IL-6 and pDC activation (**Figure 4D**). Median sCD14 was higher in all HIV-infected groups irrespective of ART timing compared to HIV negative individuals (**Figure 4E**). sCD14 plasma levels correlated with monocyte activation (rho=0.47, p=0.035; **Figure 4F**) but not to mDC or pDC activation (**Figures 4G, H**). Overall, our results suggest that ART during HIV infection, irrespective of the timing of ART initiation, may be associated with specific systemic inflammatory markers, with some of these surprisingly more elevated in individuals with ART exposure during the hyperacute phase of infection.

### Early ART Preserves pDC Cytokine Production Function

**Figure 5:**
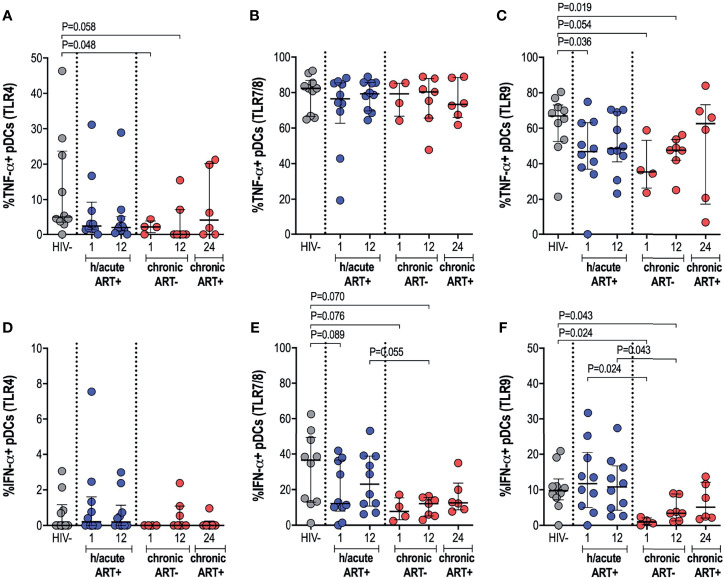
pDC cytokine production in response to TLR antigen stimulation. HIV negative individuals and hyperacute and chronic ART groups at month 1, 12 and 24 post-infection were assessed for pDC TNF-α production following stimulation of **(A)** TLR4, **(B)** TLR7/8 or **(C)** TLR9 and IFN-α production following stimulation of **(D)** TLR4, **(E)** TLR7/8 or **(F)** TLR9.

We further ascertained functional capacity of APCs by assessing cytokine production (TNF-α and IFN-α) following stimulation of TLR4, 7/8 or 9 pathways (**Supplementary Figure 4**). pDC cytokine expression capacity was impacted by the timing of ART initiation. TNF-α production by pDCs following TLR4 stimulation was reduced in the HIV infected persons not commencing immediate ART compared to uninfected controls (p=0.048 at 1-month, p=0.058 at 12-months) (**Figure 5A**). There were no changes in TNF-α production noted for the study groups following TLR7/8 stimulation of pDCs (**Figure 5B**), however, production of TNF-α following TLR9 stimulation was lower in HIV infected hyperacute treated group at 1-month (p=0.036) and in the untreated group at 1-month (p=0.054) and at 12-months (p=0.019) post-infection (**Figure 5C**). Production of IFN-α by pDCs following TLR4 stimulation was similar across the study groups (**Figure 5D**) but there was trend towards impaired IFN-α production following TLR7/8 stimulation for the HIV infected groups irrespective of ART initiation timing (**Figure 5E**). TLR9 stimulation of pDCs revealed comparable levels of IFN-α production in HIV uninfected compared to HIV infected hyperacute treated individuals but impaired production of the cytokine in late treated individuals compared to HIV uninfected and hyperacute treated persons (p=0.024 at 1-month, p=0.043 at 12-months, **Figure 5F**). No significant differences were found in median cytokine release of monocytes in ART hyperacute or chronic treated groups in comparison to HIV negative individuals (**Supplementary Figures 5A–F**). Although trends of lower TLR9-induced TNF-α were exhibited by monocytes at 1-month post-infection (p=0.063 in ART hyperacute; p=0.077 in ART chronic, **Supplementary Figure 5C**), cytokine production normalised by 12-months post-infection in both groups. IFN-α release trended lower following TLR7/8 stimulation in acute untreated infection compared to early ART individuals (p=0.076, **Supplementary Figure 5E**), but this too stabilised by 12-months post-infection. Similarly, mDC cytokine production remained unaffected by HIV infection (**Supplementary Figure 6**). There was altered TNF-α release following TLR7/8 stimulation in untreated infection (p=0.024 at 1-month, p=0.055 at 12-months; **Supplementary Figure 6B**), but this function was restored following late ART initiation.

## Discussion

Immune preservation or reconstitution and rate of disease progression following HIV infection are dependent on the timing of cART commencement, and the advantages of ART initiation in the earliest stages of HIV infection are evident. However, it is not fully understood which immune pathways are positively impacted by early ART, and detrimental or side effects of ART on host immunity have rarely been explored. Consistent with previous studies, we report that treatment during hyperacute infection results in more rapid viral suppression with minimal CD4 count loss and suppression of detrimental T cell activation (10, 17, 21). Further, we show that in patients who initated treatment after approximately 1 year infection, T cell activation stabilised to levels comparable to HIV negative controls by 1 year post-ART, consistent with previous findings for individuals who start ART during early chronic phase infection (32). We extend our analysis to explore the impact of therapy in hyperacute HIV-1 subtype C infection on APCs which play a key role in mediating innate immunity and bridging innate to adaptive immunity. ART hyperacute individuals displayed elevated monocyte and pDC frequencies longitudinally. Distribution of monocyte subsets in HIV infection were distinctly different from uninfected profiles and were relatively ART timing dependent. Phenotypic profiles of early ART groups revealed sustained activation of monocytes and pDCs; and monocyte activation strongly associated with inflammatory markers IL-6 and sCD14. Furthermore, HIV-1 induced changes in APC expression of co-stimulatory molecules involved in antigen presentation were not alleviated by either acute or chronic ART initiation. Notably, early therapy preserved pDC IFN-α responses to TLR antigenic stimulation.

We recently reported that hyperacute HIV infection was associated with altered myeloid and lymphoid blood counts even before ART could be initiated (16). We extend on these findings by longitudinal follow up of HIV infected individuals who initiated ART during Fiebig stages I-II compared with those who initiated treatment during chronic infection and report variable impact on APC frequencies, activation and function. Expansion of blood monocytes was observed in HIV infected participants with no resolution into the early chronic phase of infection in both ART treated and untreated groups thus indicating that treatment during the hyperacute phase does not correct certain immune alterations induced by HIV-1 infection. Redistribution of monocyte subsets was observed post-infection irrespective of ART timing. Previous studies indicate that ART-naïve infection is characterised by lower classical and elevated non-classical monocyte numbers (33–35). Similarly, reduced classical monocyte frequencies were observed in untreated infection, whilst early therapy preserved classical monocyte frequencies. In contrast to previous reports, non-classical monocyte frequencies were not elevated during infection which could be due to differences in demographic and clinical factors such as age, sex, ethnicity, viral subtypes and underlying comorbidities, with our cohort of 18–23-year-old females infected with HIV-1 subtype C in Durban, South Africa. Interestingly, intermediate monocytes were significantly reduced in all ART groups and may indicate unexplored effects of early ART on this immune cell subset. We also noted expansion of a previously under-reported CD14lowCD16- phenotype in all infected groups that requires further investigation. Hyperacute phase ART treated individuals also had higher pDCs frequencies, consistent with reports of increased blood pDCs following intravenous SIV infection (36–38), and individuals who started ART in acute HIV infection were observed to have increased pDC frequencies following analytic treatment interruption (ATI) even before the detection of HIV RNA (39).

Persistent immune activation and exhaustion occur concomitantly in viraemic infection (40). Here, untreated infection was associated with the loss of activation receptors on monocytes and pDCs with expression restored following late therapy initiation, whereas monocyte and pDC activation were sustained longitudinally post-infection following hyperacute treatment. Furthermore, monocyte activation associated with elevated plasma IL-6 and sCD14, and this was particularly so for sCD14 in the hyperacute ART treated group. Monocyte-linked inflammation has previously been reported subsequent to ART-mediated viral suppression in chronic infection (2, 41–43) and very early treatment (15, 16, 18). Factors linked to higher IL-6 levels in HIV infection include older age, non-black ethnicity, HIV viremia, low nadir CD4+ cell count and protease inhibitor based ART regimens (44), none of which apply to the treated groups in our study. Furthermore, sCD14, a marker of microbial translocation, was elevated in treated groups and is also indicative of ongoing immune activation (42, 45). ART improves gut immunity, but does not revert systemic microbial products to pre-infection levels (46, 47). Moreover, SIV models highlight the unavoidable deterioration of gut and lymphoid tissues even when ART is administered during AHI (48, 49). Our study thus indicates that despite treatment during hyperacute infection, HIV infected persons may already have undergone substantial gut and lymphoid tissue destruction that leads to elevated sCD14 and subsequent immunological damage.

Functionally, altered APC profiles were expected in untreated infection. HIV Nef-induced surface loss of co-stimulatory marker CD86 on APCs disrupts antigen presentation to T cells (50). Surprisingly, treatment initiated in hyperacute phase of infection did not prevent downmodulation of CD86 on APCs, rather expression resembled that of treatment-naïve infection for monocytes and mDCs. Interestingly, pDC CD86 expression was not altered in viraemic infection and lower expression levels were only observed following very early ART. Investigation of cytokines following antigenic stimulation showed no significant changes to monocyte and mDC functional capacities in untreated infection. Other studies report elevated production of TLR-induced cytokines by monocytes during untreated infection (51–53). Our study results may differ from earlier studies because the untreated participants in our study were relatively early in infection (within one-year post-infection) compared to other studies that assessed participants in late stages of HIV infection. A small sample size is also a potential limitation in our study. Notably, in the current study, pDCs displayed diminished cytokine secretion in untreated infection that was similar to previous reports (54, 55), that slightly improved after chronic phase ART, whereas hyperacute treatment preserved pDC responsiveness. The results thus suggest differential impact of ART initiated in hyperacute HIV infection phase on APC functional capacity.

The results highlight the positive effects of early ART in promoting pDC immune maintenance that may prove essential during ATI strategies. Mitchell et al. recently characterised pDC dynamics following ATI in AHI treated individuals that concur with our observations of increased frequencies of partially activated pDCs and our previous report of blunted plasma IFN-α following ART in AHI (16), which were further demonstrated as necessary for an effective early antiviral immune response (39). However, antigen presentation between innate and adaptive immunity may be sub-optimal despite ART administration at the earliest stages of HIV infection. Whether these findings suggest compromised cellular interaction or a delayed response that beneficially conserves adaptive immunity requires further examination. Furthermore, monocyte-induced systemic inflammation was not effectively halted following early ART and could explain the higher risk of comorbidities that persist in cART adhering HIV infected individuals (56, 57). Further investigations are needed to specifically identify specific immune targets or pathways that remain defective despite very early initiation of ART in HIV infected individuals or defects that may be induced by ART itself.

Limitations of this study include a small sample size, limited number of PBMCs for analysis, a short duration of longitudinal follow-up and an all female cohort. These limitations call for caution in the interpretation of our data and make it difficult to generalise our findings. For example, limited number of PBMCs resulted in low gated pDC counts and also prevented inclusion of markers such as BDCA-2 and BDCA-4 that define pDCs or BDCA-1 and BDCA-3 that identify mDCs (58). Moreover, marked sex differences in pDC responses to HIV-1 have been reported (59) and future investigations will therefore need to include HIV-infected men. Nevertheless, availability of longitudinal samples from young African females who initiated treatment during hyperacute and chronic infection allowed us to explore potential immune alterations in a population group where potential adjunctive therapy to ART may result in great public health benefit given the high burden of infection. Our study confirms that early therapy forestalls detrimental immune activation and inflammation of adaptive immune cells. It also suggests a heterogenous short- and long-term impact on the phenotype and functional characteristics of antigen presenting cells. Specifically, very early therapy was associated with preservation of pDC functions. However, despite treatment during the hyperacute phase of infection, we observed persistent monocyte activation and inflammation, plausibly a result of irreversible microbial translocation. The consequences of these alterations on host immunity and clinical outcomes warrant further investigation to identify therapeutic interventions that complement the benefits of antiretroviral therapy.

## Data Availability Statement

The original contributions presented in the study are included in the article/**Supplementary Material**. Further inquiries can be directed to the corresponding author.

## Ethics Statement

The studies involving human participants were reviewed and approved by The Biomedical Research Ethics Committee of the University of KwaZulu-Natal and the Institutional Review Board for Massachusetts General Hospital. The patients/participants provided their written informed consent to participate in this study.

## Author Contributions

KN: study design, acquisition of data, analysis of the data, interpretation of data, and drafting the manuscript. ON: study design, acquisition of data, and interpretation of data. NI and KD: acquisition of data. TN: conception, study design, supervisory support, interpretation of data, and revising the manuscript. All authors contributed to the article and approved the submitted version.

## Funding

This work was funded through a DFG German-African Network grant (grant number AL 1043/6-1). The work was also supported by the South African Department of Science and Innovation through the National Research Foundation (South African Research Chairs Initiative), the Bill and Melinda Gates Foundation (grant number INV-009283), the International AIDS Vaccine Initiative (UKZNRSA1001), the NIAID (R37AI067073) and the Victor Daitz Foundation. Additional funding was provided by the Sub-Saharan African Network for TB/HIV Research Excellence (SANTHE), a DELTAS Africa Initiative [grant # DEL-15-006]. The DELTAS Africa Initiative is an independent funding scheme of the African Academy of Sciences (AAS)’s Alliance for Accelerating Excellence in Science in Africa (AESA) and supported by the New Partnership for Africa’s Development Planning and Coordinating Agency (NEPAD Agency) with funding from the Wellcome Trust [grant # 107752/Z/15/Z] and the UK government.

## Author Disclaimer

The views expressed in this publication are those of the author(s) and not necessarily those of AAS, NEPAD Agency, Wellcome Trust, or the UK government.

## Conflict of Interest

The authors declare that the research was conducted in the absence of any commercial or financial relationships that could be construed as a potential conflict of interest.

## Publisher’s Note

All claims expressed in this article are solely those of the authors and do not necessarily represent those of their affiliated organizations, or those of the publisher, the editors and the reviewers. Any product that may be evaluated in this article, or claim that may be made by its manufacturer, is not guaranteed or endorsed by the publisher.
