# Supplementary material for: Antigen Presenting Cells Contribute to Persistent Immune Activation Despite Antiretroviral Therapy Initiation During Hyperacute HIV-1 Infection
Source: Front Immunol. 2021 Sep 24;12:738743. doi: 10.3389/fimmu.2021.738743 (PMC8498034; doi:10.3389/fimmu.2021.738743)
Supplement: Supplementary file 1 [file DataSheet_1.docx]

Supplementary Material

# Supplementary Figures

# 1.1 Supplementary Figure 1


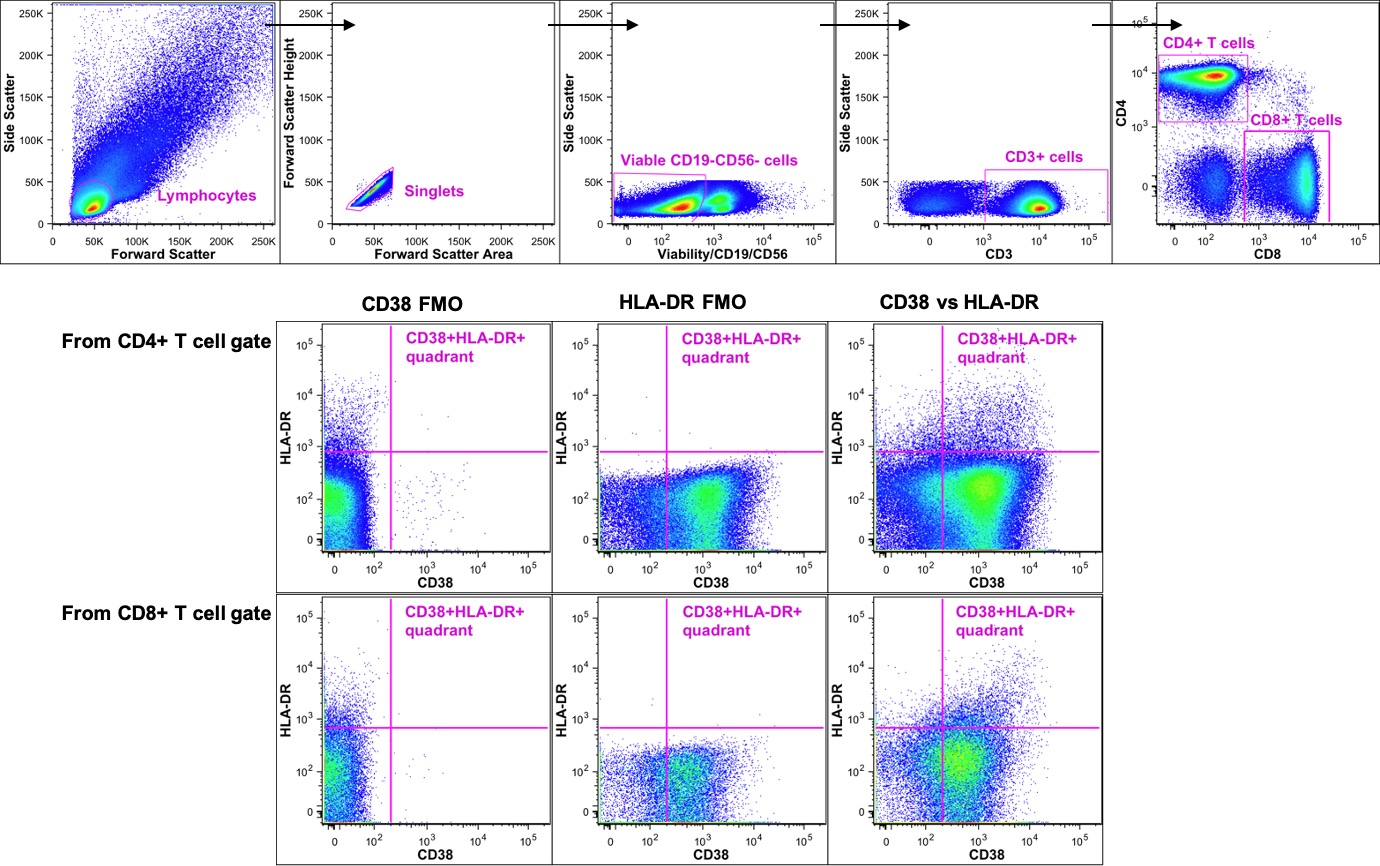


**Supplementary Figure 1: Representative gating strategy for T cells.** Side scatter area vs forward scatter area plots were used to identify lymphocytes followed by exclusion of doublets by forward scatter height vs forward scatter area. Lymphocytes were stained with viability dye, anti-CD19 and anti-CD56 to exclude non-viable cells, B cells and NK cells respectively. Anti-CD3 staining was used for T cell gating followed by identification of CD4+ and CD8+ T cells. CD4+ and CD8+ T cell activation was identified as CD38+HLA-DR+ expression by applying quadrant gating from FMO controls.

# 1.2 Supplementary Figure 2


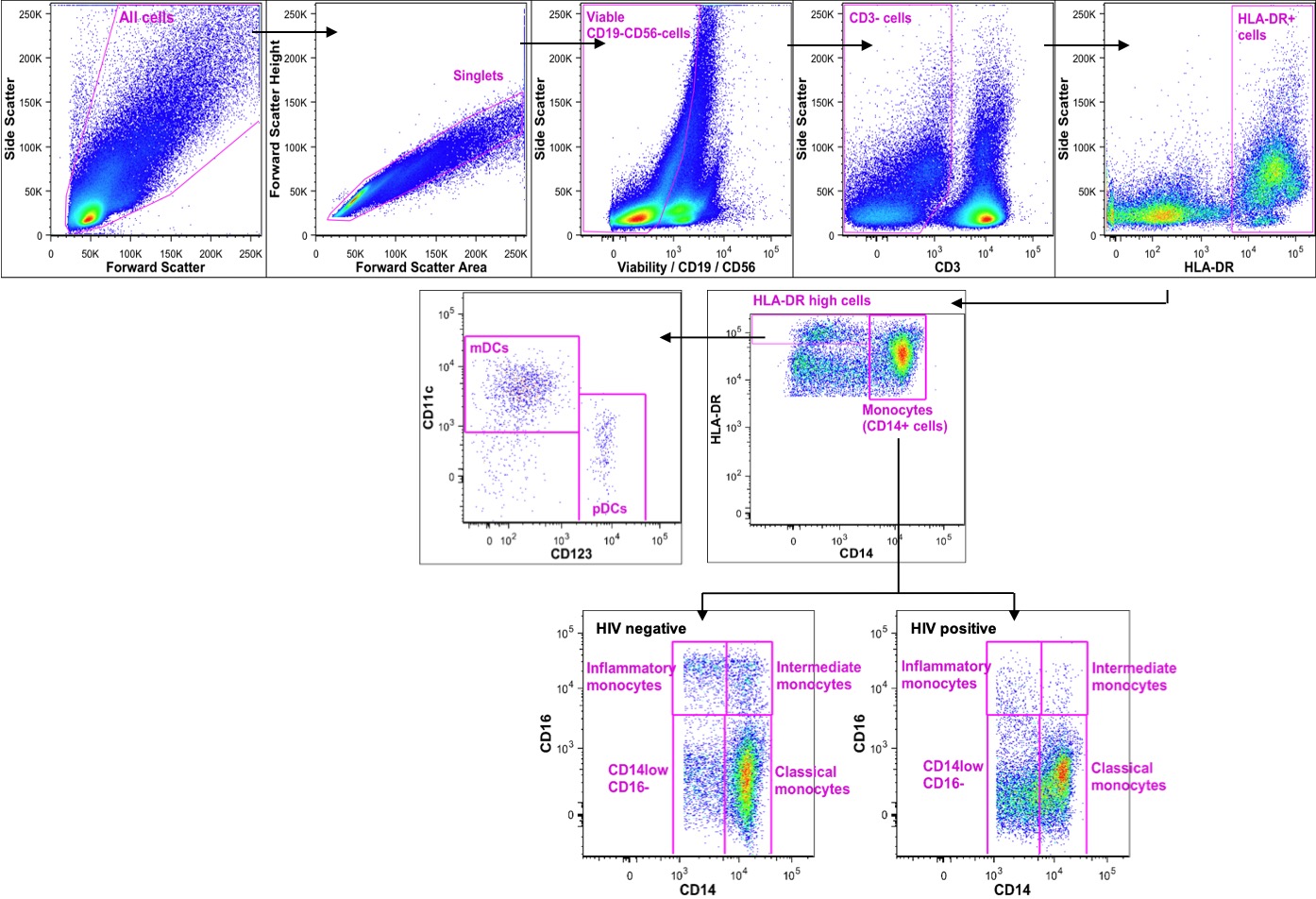


**Supplementary Figure 2: Representative gating strategy for antigen presenting cells.** Side scatter area vs forward scatter area plots were used to identify total cells followed by exclusion of doublets by forward scatter height vs forward scatter area. Total cells were stained with viability dye, anti-CD19 and anti-CD56 to exclude non-viable cells, B cells and NK cells respectively, before gating on CD3- and HLA-DR+ cells. Monocytes were identified as CD14+ and were further classified as classical, intermediate, inflammatory or CD14lowCD16- by relative expression of CD14 and CD16. mDCs and pDCs were identified as HLA-DR high cells and either CD11c+ or CD123+ respectively.

# 1.3 Supplementary Figure 3


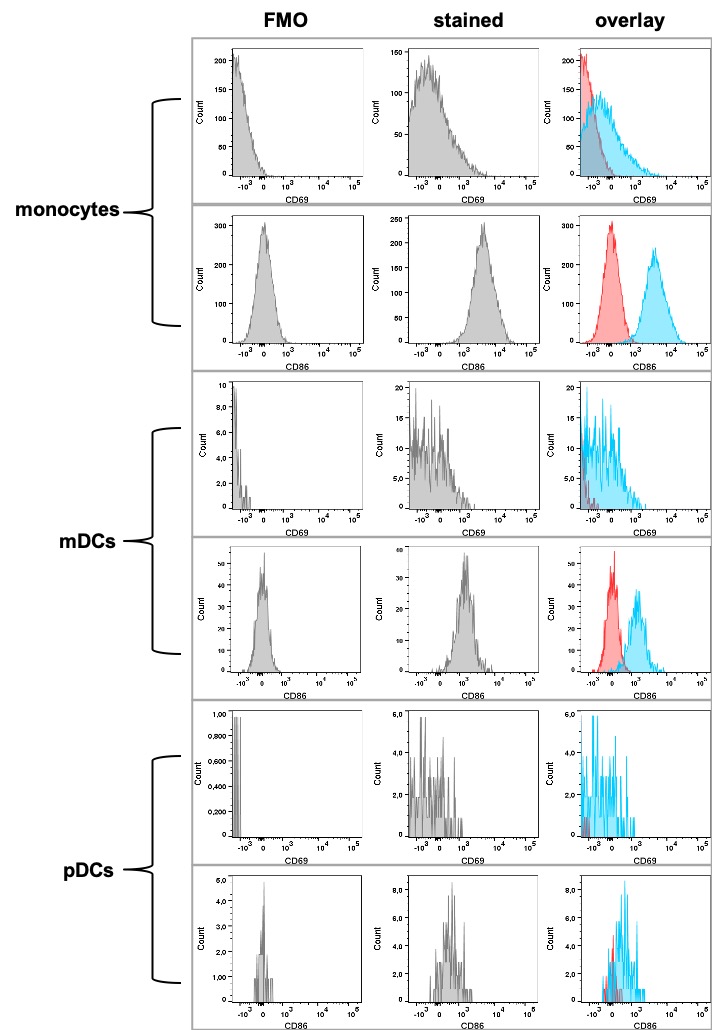


**Supplementary Figure 3: Representative histograms used for CD69 and CD86 MFI analyses.** Monocytes, mDCs and pDCs were assessed for CD69 and CD86 expression using FMO controls and stained samples. Overlays show differences in MFI between FMO (red) and stained sample (blue).

# 1.4 Supplementary Figure 4


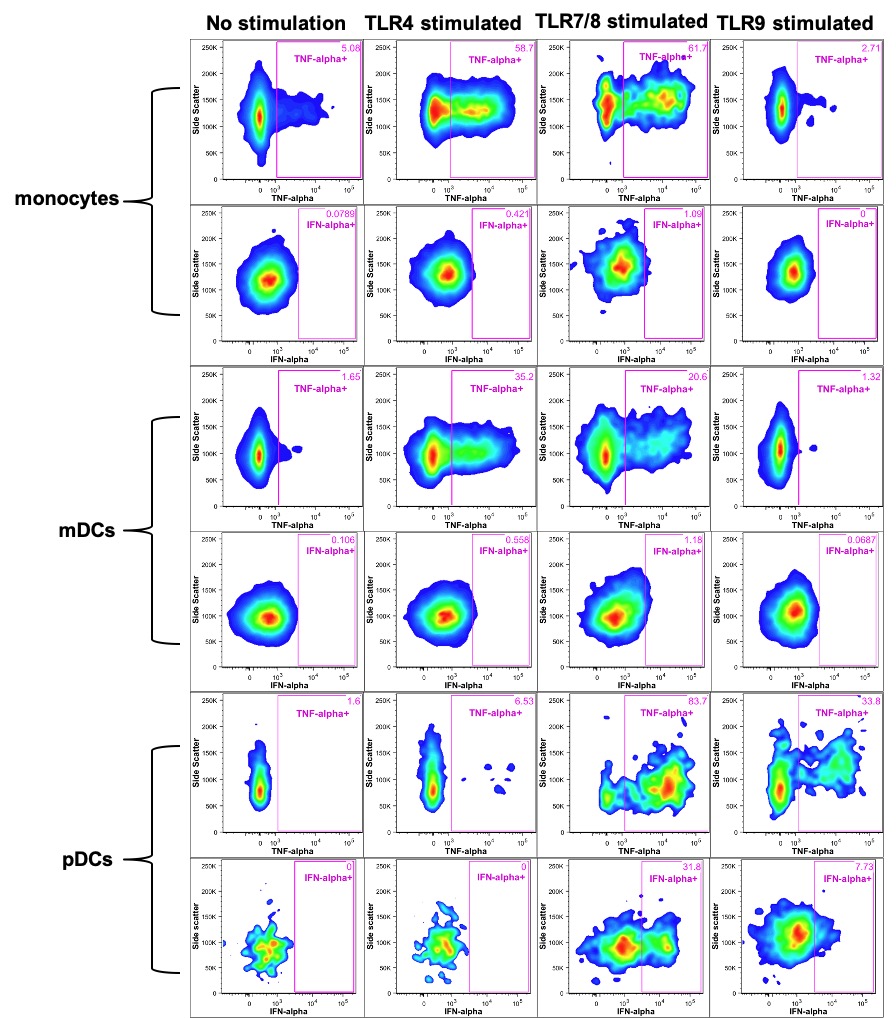


**Supplementary Figure 4: Representative gating strategy used for cytokine production analyses in response to TLR antigen stimulation.** Monocytes, mDCs and pDCs were assessed for TNF-α and IFN-α production following stimulation with TLR4, TLR7/8 or TLR9 agonist. No stimulation conditions were used to subtract background from stimulated conditions.

**1.5 Supplementary Figure 5**

**
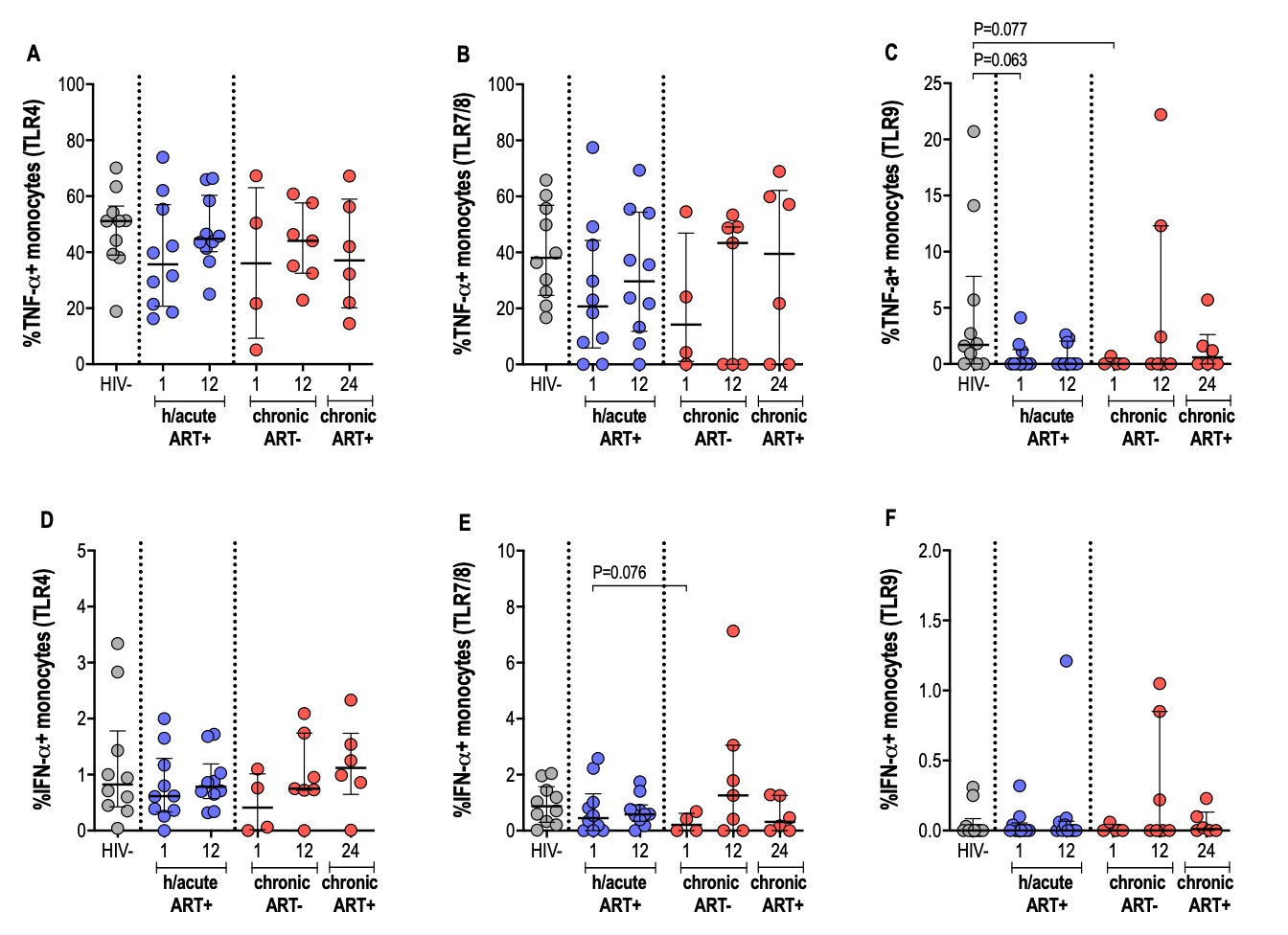
**

**Supplementary Figure 5: Monocyte cytokine production in response to TLR antigen stimulation.** HIV negative individuals and hyperacute and chronic ART groups at month 1, 12 and 24 post-infection were assessed for monocyte TNF-α production following stimulation of **(A)** TLR4, **(B)** TLR7/8 or **(C)** TLR9 and IFN-α production following stimulation of **(D)** TLR4, **(E)** TLR7/8 or **(F)** TLR9.

**1.6 Supplementary Figure 6**

**
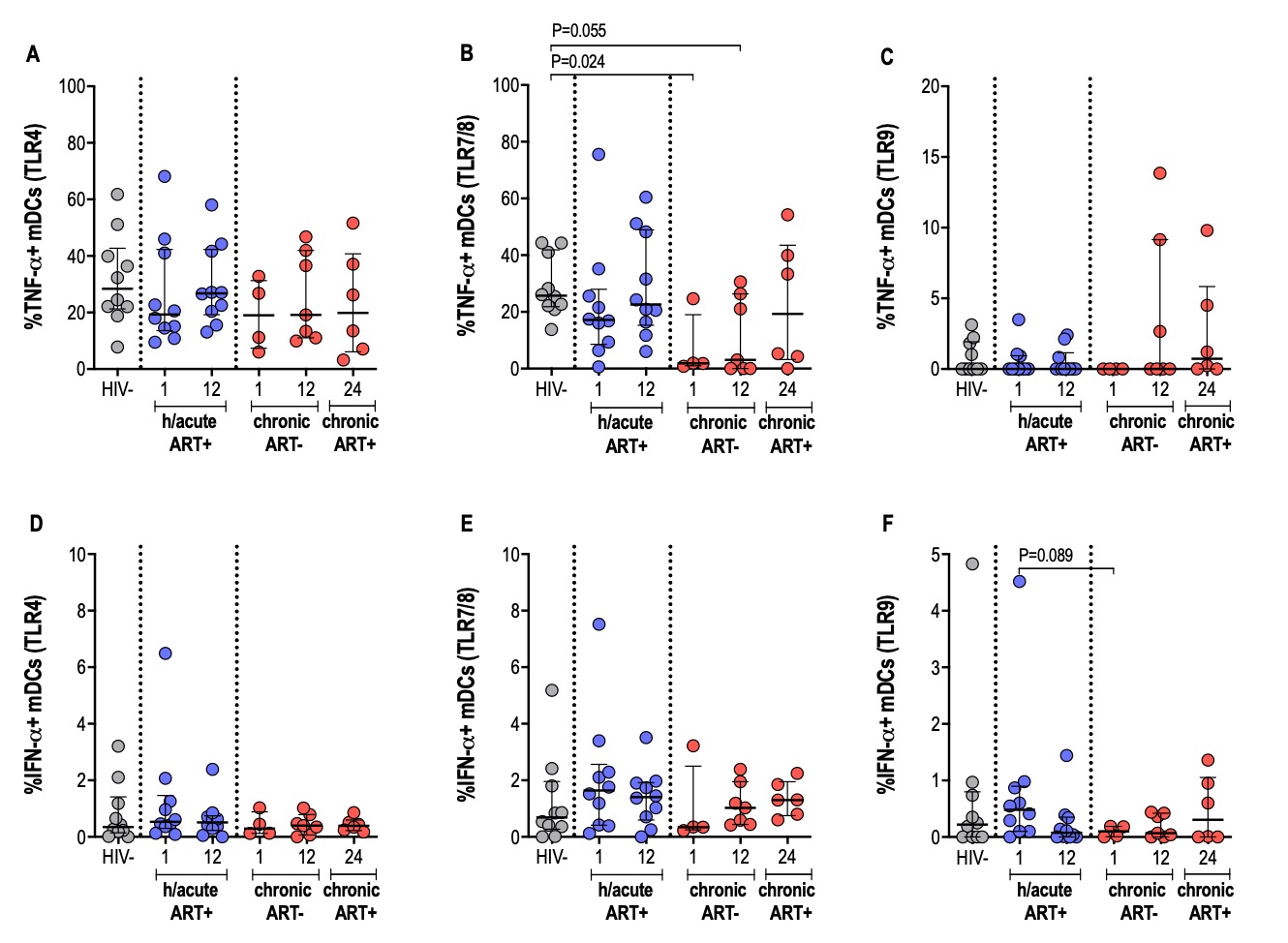
**

**Supplementary Figure 6: mDC cytokine production in response to TLR antigen stimulation.** HIV negative individuals and hyperacute and chronic ART groups at month 1, 12 and 24 post-infection were assessed for mDC TNF-α production following stimulation of **(A)** TLR4, **(B)** TLR7/8 or **(C)** TLR9 and IFN-α production following stimulation of **(D)** TLR4, **(E)** TLR7/8 or **(F)** TLR9.
